# Immediate Reversal of Dabigatran by Idarucizumab Prior to Laboratory and Imaging Results in Acute Stroke

**DOI:** 10.3389/fneur.2019.00230

**Published:** 2019-03-15

**Authors:** Maren Hieber, Juergen Bardutzky

**Affiliations:** Department of Neurology and Neurophysiology, Medical Center, University of Freiburg, Freiburg, Germany

**Keywords:** dabigatran, idarucizumab, intravenous thrombolysis, door-to-needle time, case report

## Abstract

We report a case of intravenous thrombolysis in acute ischemic stroke of anterior choroidal artery following the antagonization of dabigatran with idarucizumab. No secondary complication, like hemorrhagic or thrombotic/thrombembolic event, of neither idarucizumab nor subsequent intravenous thrombolysis emerged. The recent approval of idarucizumab enables intravenous thrombolysis despite preexisiting oral anticoagulation with dabigatran, but raises the question of the optimal management and work flow of patients under medication with dabigatran and with acute neurological deficit, highly suspicious for an acute cerebrovascular event. In contrast to hitherto case reports and series, here, we explicitly refrained from awaiting the results of the thrombin time, as a marker for present anticoagulation by dabigatran, as well as the results of cerebral imaging before administration of idarucizumab. Based on the presented case we propose this approach to minimize door-to-needle time of intravenous thrombolysis in acute ischemic stroke and thus to enhance the chance for a good outcome.

## Background

Dabigatran, a direct thrombin inhibitor, is approved for treatment and secondary prevention of venous thromboembolism, prevention of stroke, and systemic embolism in non-valvular atrial fibrillation and prevention of venous thromboembolism upon knee and hip replacement surgery (1).

Thrombin clotting time (TT) is a common and accessible method for measuring the anticoagulant effects of dabigatran (2).

Idarucizumab is a humanized monoclonal antibody fragment with 350 times higher binding affinity for dabigatran. It is approved since 2015 for antagonization of the anticoagulant effects of dabigatran in case of life-threatening hemorrhage or urgently indicated operations and interventions (3).

Until recently intravenous thrombolysis with recombinant tissue plasminogen activator (rtPA) in acute ischemic stroke in patients under medication with dabigatran was not possible since present anticoagulation displays a contraindication for rtPA. Following as well American as German guidelines intravenous thrombolysis could only be considered if laboratory tests are normal or time from last intake is more than 48 h (4, 5). The recent approval of idarucizumab now allows the fast antagonization of dabigatran and thereby normalization of coagulation within minutes. The scenario of dabigatran reversal and subsequent intravenous thrombolysis in case of acute ischemic stroke was stated as in-label use of both, idarucizumab and recombinant tissue plasminogen activator (6, 7), but is not covered by the current guidelines. Despite exclusion of this indication in the original pivotal study of idarucizumab (3), several recent case reports and series provide increasing evidence, that reversal of dabigatran by idarucizumab followed by intravenous thrombolysis in acute ischemic stroke is effective and safe (8, 9). However, large randomized controlled trials on this topic are lacking, and the optimal management and diagnostic work flow of this approach is still under discussion (10, 11).

The WAKE-UP trial evaluated the safety and effectiveness of MRI-based intravenous thrombolysis in acute ischemic stroke patients with unknown time of onset. The time from last known well to possible thrombolysis had to be >4.5 h, since otherwise patients would have fulfilled the standard eligibility criteria for intravenous thrombolysis. Patients were randomized, when the MRI revealed an acute ischemic lesion on the diffusion-weighted imaging (DWI) and no correspondent hyperintense lesion on the fluid-attenuated inversion recovery (FLAIR) sequences, the latter defining the DWI-FLAIR-mismatch. The trial showed a significantly better functional outcome in patients with acute ischemic stroke with unknown time of onset and proven DWI-FLAIR-mismatch, that received intravenous thrombolysis compared to placebo (12).

Here, we report the case of a patient under medication with dabigatran with acute ischemic stroke, who received idarucizumab and subsequently intravenous thrombolysis. In contrast to formerly reported cases (11, 13, 14), here, we explicitly refrained from awaiting the results of (i) the thrombin time (TT), as a marker for present anticoagulation by dabigatran and (ii) the cerebral imaging before administration of idarucizumab to minimize door-to-needle time and thus to enhance the chance for a good outcome. The decision for the application of idarucizumab was based on anamnesis and clinical examination only. For intravenous thrombolysis in turn the emergency cerebral imaging was considered for sure.

## Case Presentation

**Figure 1:**
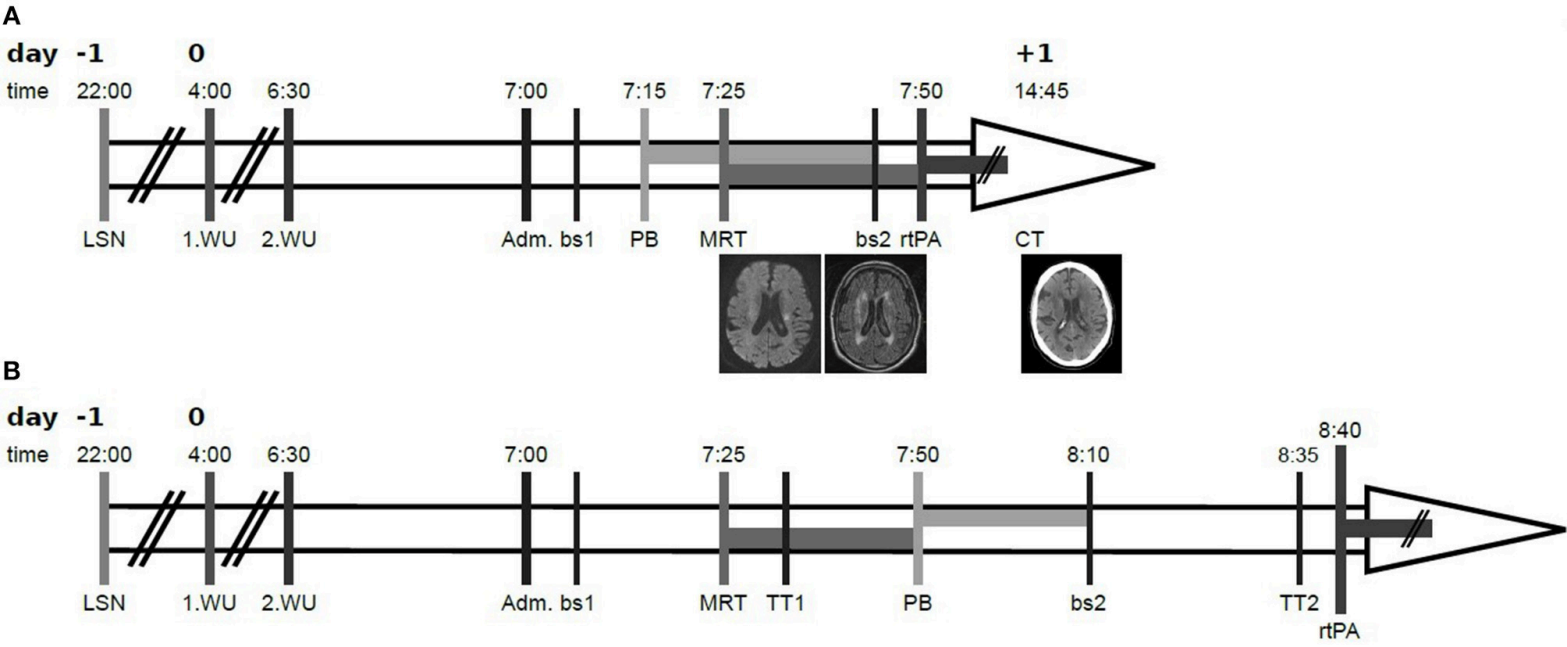
Time course of the reported case. **(A)** shows the actual course, **(B)** shows the hypothetical course in case of awaiting laboratory and imaging results. All above the number of day is stated, relative to day 0 as admission day, followed by exact time in the second row. Time of last seen well (LSN) was reported for the eve of (day −1. 22:00). First wake up (1.WU) with neurological deficit was reported for 4:00, second wake up (2.WU) for 6:30. The patient was admitted (Adm.) at 7:00 and clinically examined. In parallel a first blood sample was taken (bs1), followed by the administration of idarucizumab (PB), cerebral imaging (MRI), a second blood sample (bs2), and intravenous thrombolysis (rtPA). Analyses of the blood samples took 25–30min, so results for Thrombin time (TT1 and TT2, respectively) were available after this time. Exemplary slices of the diffusion weighted imaging (left) and fluid-attenuated inversion recovery (middle) sequences at admission as well as the control CT at day 1 (right) are shown in the last row of **(A)**. By comparing the actual starting point of intravenous thrombolysis (7:50) with the hypothetical one (8:40), where idarucizumab is applied after receiving the results of the first blood sample and after cerebral imaging, the reduction of the door-to-needle time is comprehensible.

An 81-year old man was admitted at 7:00 a.m. to our hospital due to wake up symptoms of right-sided hemiparesis and dysarthria. Timepoint of last known well was stated for the eve of (22:00 p.m.). The patient reported to had woken up at 4 o'clock in the morning noticing a paresis of the right leg, but had fallen asleep again. At 6:30 a.m. he had woken up again with right-sided hemiparesis and dysarthria, whereupon the emergency medical services had been called (see Figure 1 for a graphical presentation of the time course). Clinical examination at admission revealed a moderate right-sided sensomotoric hemisyndrome with dysarthria and inconsistent signs of neglect [National Institutes of Health Stroke Scale (NIHSS) = 6 points]. Following the house-intern standard operating procedure the patient was admitted and clinically evaluated outside of the imaging room. As a matter of routine, the results of this emergency evaluation determine the subsequent procedure, especially the choice of imaging modality.

Due to permanent atrial fibrillation the patient was taking dabigatran 150 mg twice per day. Last intake was reported distinctly for the eve of, i.e., ~12 h prior to admission.

Based on the assured intake of dabigatran ~12 h ago and the reasonable suspicion of an acute cerebrovascular event, i.e., either cerebral ischemia or intracerebral hemorrhage, idarucizumab 2 × 2.5 g was applied immediately upon neurological examination at admission, prior to cerebral imaging as well as prior to proven (by laboratory testing, i.e., TT) anticoagulation. Due to unknown time of symptom onset and mild to moderate symptoms of acute cerebrovascular event we decided to perform primary MRI (according to the house-intern standard operating procedure, and with respect to the WAKE-UP trial (12). MRI-DWI sequences revealed an acute ischemia in the area of the left anterior choroidal artery without correspondent hyperintensity on the FLAIR-sequences (see Figure 1). No occlusion of any cerebral artery was shown in MRI. After MRI and completed application of idarucizumab a new blood sample was taken, and intravenous thrombolysis with 90 mg alteplase was started immediately, resulting in a door-to-needle time of 50 min. During ongoing application of intravenous thrombolysis, the result of second TT (of blood sample taken directly upon completion of idarucizumab application) was examined. Since TT revealed normalized here (TT_post Idarucizumab_ = 18.3 s, TT_initial_ = 61.7 s), application of rtPA was proceeded to the full dose of 90 mg (equivalent 0.9 mg/kg body weight). Follow up CT on the next day revealed a small demarcated ischemia in the corona radiata (see Figure 1), corresponding to the aforementioned diffusion-restricted area, without intracranial hemorrhage. Dabigatran was restarted in the evening of day 4 (i.e., 108 h upon thrombolysis). The patient was discharged on day 26 with mildly ameliorated hemiparesis (NIHSS = 4 points).

## Discussion

Here, we present a case of intravenous thrombolysis in acute ischemic stroke in the territory of the left anterior choroidal artery following the application of idarucizumab in a patient taking dabigatran. Whereas, a higher risk for progression in infarctions of anterior choroidal artery compared to hemispheric infarcts had been shown (15–17), no differences regarding the rate of poor outcome was shown (15). Since occlusions of the anterior choroidal artery are not accessible for mechanical thrombectomy, intravenous thrombolysis remains the only acute therapeutic option in such patients, that reduced the risk of stroke progress and improved functional outcome (16).

In the presented case application of idarucizumab was effective and safe, since immediate normalization of coagulation was proven by laboratory results—albeit checked only in retrospect—and no thrombotic or allergic event occurred. Subsequent intravenous thrombolysis was moderately effective and safe, since the patient's symptoms improved from NIHSS of 6 to 4 points and no complication, like hemorrhagic or allergic event occurred. This is in line with the mainly positive results of former case reports and series reporting good outcome and lack of complications. Nevertheless, the pivotal study of idarucizumab reported thrombotic events in 6–7% of patients treated with idarucizumab, but considered these events more likely to reflect the underlying prothrombotic state than to be a direct effect of idarucizumab (3). Further reports on thrombotic events span a case of deep vein thrombosis and bilateral pulmonary embolism 5 days after application of idarucizumab and intravenous thrombolysis (7) and a severe second infarction (18). In summary, thrombotic events following the application of idarucizumab seem to be rare, and are not proven to be induced by idarucizumab, but should be considered possible.

Regarding the oppositional complication of an intracerebral hemorrhage following intravenous thrombolysis only minutes after still present anticoagulation a recent review analyzed the safety and effectiveness of intravenous thrombolysis in patients taking non-vitamin K oral anticoagulants (NOACs). Even if limited on anecdotal experiences of case reports and series this review provides further evidence that patients taking NOACs in general might not have an increased bleeding risk upon intravenous thrombolysis. With respect to dabigatran only, authors found, that patients treated with idarucizumab were significantly more likely to have favorable outcomes and are numerically less likely to bleed and die (19). The here presented case is another one without hemorrhagic complication.

With respect to the specific management of patients taking dabigatran and suspicious for an acute cerebrovascular event and in contrast to former case reports on the reversal of dabigatran by idarucizumab followed by intravenous thrombolysis in acute ischemic stroke we present new important practical aspects:

Results of (i) laboratory testing (i.e., of TT in the majority of cases) and of (ii) cerebral imaging were not awaited before administration of idarucizumab. Rather, clinical decisions, consisting in application of idarucizumab and rtPA, were based on anamnesis, clinical examination and—with respect to application of rtPA only—results of cerebral imaging. Rationale for this approach is the high likelihood, that the patient's acute neurological deficit of dysarthria and hemiparesis is caused by an acute cerebrovascular disease, i.e., cerebral ischemia or intracerebral hemorrhage (ICH). Both will benefit from the antagonization of the anticoagulant effects of dabigatran: the latter due to its hemorrhagic nature, the former due to the resulting possibility of intravenous thrombolysis. By starting idarucizumab infusion immediately after admission, before laboratory results and imaging, idarucizumab infusion (which takes 20–30 min) was finished during ongoing cerebral imaging. In the reported case, ischemia was proven by DWI, and decision for thrombolysis was based on DWI-FLAIR mismatch in dependence on the WAKE-UP trial (12). By meanwhile completed infusion of idarucizumab, we were able to start intravenous thrombolysis immediately afterwards. In the hypothetical alternative case of an ICH the risk of ongoing or re-bleeding would have been minimized and/or any potential surgical or interventional procedure would have been enabled as fast as possible by completed antagonization of dabigatran. Subarachnoidal hemorrhage, a further possible differential diagnosis, can be clinically excluded by headache and/or meningism, and—anyway—should be treated with idarucizumab as well. In summary, we consider the exact specification of an acute cerebrovascular event of secondary importance, after the most important aspect of a fast antagonization of dabigatran. With respect to acute ischemic stroke only it remains the question if patients with large vessel occlusion (LVO) and those without should be treated in a different way. Such differentiation is proposed by Diener et al. (20), who recommend in case of LVO accessible for mechanical thrombectomy to perform only the latter, without preceding intravenous thrombolysis upon dabigatran reversal. Our proposed approach contradicts this recommendation in the sense, that following our approach the differentiation between LVO and no LVO would be done after—or during ongoing—application of idarucizumab. With respect to the not awaited laboratory results to ensure a present anticoagulation by dabigatran, we consider the safety profile of idarucizumab good enough (3, 7) to administer it even in case of only anamnestic intake of dabigatran.

Even in stroke mimics (e.g., seizure, migraine) application of idarucizumab is defensible, since it may not lead to severe side effects, as idarucizumab has neither assured pro- nor anticoagulant effects besides dabigatran antagonization (3). Furthermore,—if necessary—next regular dabigatran dose can be administered as scheduled.

Especially in case of cerebral ischemia the main aim for the organization of acute stroke patients is the biggest possible celerity, i.e., to minimize the door-to-needle time and thereby to enhance the chance for a good outcome (21). Every procedure of patient's admission, like anamnesis, clinical examination, measuring the anticoagulant effects of dabigatran, imaging acquisition, infusion time of idarucizumab, takes time, and delays the start of thrombolysis. Thus, parallelization of the single procedures is desirable. Here, we met this target by premature application of idarucizumab, and thereby parallelization of transport to and accomplishment of cerebral imaging as well as laboratory, i.e., TT measurement with idarucizumab infusion.

In summary, we consider this approach to be not only justifiable at least in cases, where recent intake of dabigatran is reliably reported and clinical symptoms and anamnesis yields in a high probability of either cerebral ischemic or hemorrhagic stroke, but also advantageous for the patient's outcome.

To encourage—or to discard—the proposed approach prospective studies and clinical trials on this topic, evaluating not only the general feasibility but also the effect on patients' outcome are needed.

## Data Availability

All datasets generated for this study are included in the manuscript.

## Ethics Statement

No investigations or interventions were performed outside routine clinical care for this patient. Since this is a case report, without experimental intervention, formal research ethics approval is not required. Written informed consent for publication was obtained from the patient.

## Author Contributions

MH wrote and edited the manuscript. JB reviewed and edited the manuscript.

### Conflict of Interest Statement

The authors declare that the research was conducted in the absence of any commercial or financial relationships that could be construed as a potential conflict of interest.
